# Neuronal activity modulates the expression of secretagogin, a Ca^2+^ sensor protein, during mammalian forebrain development

**DOI:** 10.1111/apha.70031

**Published:** 2025-03-31

**Authors:** János Hanics, Evgenii O. Tretiakov, Roman A. Romanov, Anna Gáspárdy, Zsófia Hevesi, Robert Schnell, Tibor Harkany, Alán Alpár

**Affiliations:** ^1^ Department of Anatomy Semmelweis University Budapest Hungary; ^2^ SE NAP Research Group of Experimental Neuroanatomy and Developmental Biology Semmelweis University Budapest Hungary; ^3^ Department of Molecular Neurosciences Center for Brain Research, Medical University of Vienna Vienna Austria; ^4^ Department of Neuroscience Biomedicum 7D, Karolinska Institutet Solna Sweden

**Keywords:** calcium‐binding protein, light deprivation, neurodevelopmental disorder, single‐cell transcriptomics, trisomy

## Abstract

**Aim:**

Because of their stable expression, some EF‐hand Ca^2+^‐binding proteins are broadly used as histochemical markers of neurons in the nervous system. Secretagogin is a member of “neuron‐specific” Ca^2+^‐sensor proteins, yet variations in its expression due, chiefly, to neuronal activity remain ambiguous. We aimed to fill this gap of knowledge both in its use as a cell identity marker and for mechanistic analysis.

**Methods:**

We mapped secretagogin distribution in human foetal forebrains. Then, *Scgn*‐iCre::Ai9 mice in conjunction with single‐cell RNA‐seq were used to molecularly characterize cortical secretagogin‐expressing neurons. Besides the in vitro manipulation of both SH‐SY5Y neuroblastoma cells and primary cortical cultures from foetal mice, the activity dependence of secretagogin expression was also studied upon systemic kainate administration and dark rearing.

**Results:**

In the mammalian brain, including humans, both transient and stable secretagogin expression sites exist. In the cerebral cortex, we identified deep‐layer pyramidal neurons with lifelong expression of secretagogin. Secretagogin expression was affected by neuronal activity: it was delayed in a cohort of human foetuses with Down's syndrome relative to age‐matched controls. In mice, dark rearing reduced secretagogin expression in the superior colliculus, a midbrain structure whose development is dependent on topographic visual inputs. In contrast, excitation by both KCl exposure of SH‐SY5Y cells and primary cortical neurons in vitro and through systemic kainate administration in mice increased secretagogin expression.

**Conclusion:**

We suggest that secretagogin expression in neurons is developmentally regulated and activity dependent.

## Graphical abstract

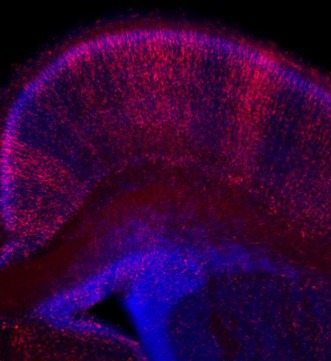

## 1 INTRODUCTION

Ca^2+^‐sensor proteins are critical to regulating neuronal differentiation, intercellular communication, resilience, and survival.^1^, ^2^, ^3^ Apart from calmodulins and calcyclins that are required for cell‐cycle control and the proliferation of neural progenitors, neuron‐specific EF‐hand Ca^2+^‐binding proteins (e.g., parvalbumin, calbindin‐D28k, calretinin) are recognized as molecular constituents of postnatal, mature neurons of the vertebrate brain.^4^, ^5^ This is because their function is chiefly relevant to the modulation of cellular excitability and the maintenance of synaptic neurotransmission,^6^, ^7^, ^8^ mechanisms fundamentally reliant on the spatial and temporal regulation of intracellular Ca^2+^ signaling in all organisms with a nervous system.

Secretagogin (gene: *Scgn*; protein: SCGN) is an atypical Ca^2+^‐sensor protein because the onset of its expression coincides with the generation of the first neuroblasts in many brain regions during development.^9^, ^10^ Moreover, major phylogenetic differences exist in the size and identity of neuronal contingents harboring secretagogin expression between rodent and primate brains.^9^, ^11^, ^12^, ^13^ Particularly, migrating neurons in the superficial layers of the cerebral cortex can transiently express secretagogin in primates/humans but not in mice.^14^ Ectopic secretagogin overexpression in gamma‐aminobutyric acid (GABA) interneurons of the mouse neocortex suggests a role for this protein in dendritogenesis and synapse formation.^14^ Thus, secretagogin could qualify as a neuron‐specific Ca^2+^‐sensor protein that undergoes activity‐dependent expression rather than being a stable marker of cell identity. Nevertheless, this hypothesis could benefit from in vivo testing in both physiology and disease. At the same time, the molecular identity of neuronal contingents destined for deep cortical layers and whose secretagogin expression is stable throughout life has not been studied earlier.

Here, we map secretagogin expression during pre‐ and post‐natal development in human brains, including transient and stable neuronal *Scgn* expression sites. Subsequently, we demonstrate a developmental delay in *Scgn* expression in Down's syndrome. We then use single‐cell RNA‐seq in *Scgn*‐iCre::Ai9 reporter mice to show that deep‐layer neurons with stable *Scgn* expression are glutamatergic and exhibit molecular signatures of corticothalamic projection neurons. These neurons are placed remarkably uniformly, ~200–300 μm apart from one another in the neocortex. Activity‐dependent *Scgn* expression is supported by increased protein levels upon systemic kainate application, as well as KCl exposure of primary neurons in vitro. In contrast, visual deprivation is associated with reduced expression of this Ca^2+^‐sensor protein. Cumulatively, we offer a broader hypothesis on the activity‐dependent use of secretagogin for the exocytosis of neurotransmitters and hormones, as intercellular communication is shaped in the developing nervous system.

## 2 METHODS

### 2.1 Human foetuses, tissue preparation, and quantitative histochemistry

Neuroanatomy was performed in foetal control brains from *n* = 12 male and *n* = 9 female subjects, as well as *n* = 6 with unknown sex, with normal development between gestational weeks (GWs).^14^, ^15^, ^16^, ^17^, ^18^, ^19^, ^20^, ^21^, ^22^, ^23^, ^24^, ^25^, ^26^, ^27^, ^28^, ^29^, ^30^, ^31^, ^32^, ^33^, ^34^ Another *n* = 13 male, *n* = 7 female, and *n* = 2 foetal brains with unknown sex, but all with Down's syndrome, were also included. We further included samples obtained after birth (*n* = 3; Table S1). Foetal brain tissues were obtained from spontaneous or elective abortions and used in compliance with the Declaration of Helsinki and provided for analysis by the Brain Bank of the Institute of Neurology, Medical University of Vienna, Austria, with their use for histopathology approved by the Human Ethical Committee of the Medical University of Vienna (no. 104/2009).^15^, ^16^ Only cases without genetic disorders, head injury, neurological complications, chromosomal aberrations, or *post‐mortem* autolysis were included as controls. In all cases, neuropathological examination excluded major central nervous system malformations, severe hypoxic/ischemic encephalopathy, intraventricular haemorrhage, severe hydrocephalus, meningitis, or ventriculitis.

Three micrometer‐thick sections prepared from formalin‐fixed, paraffin‐embedded tissue blocks were mounted onto pre‐coated glass slides (StarFrost), deparaffinized and rehydrated. Sections were pre‐treated in low‐pH EnVision FLEX antigen retrieval solution at 98°C for 20 min (PTLink; Dako) and then exposed to a polyclonal anti‐secretagogin antibody made in rabbit (gifted by Ludwig Wagner, 1:10 000^17^; Table S2). A biotinylated anti‐rabbit secondary antibody produced in donkey (K5007, Thermo Fisher) and the DAKO EnVision detection kit including peroxidase/3,3‐diaminobenzidine‐tetrahydrochloride (Agilent) were used to visualize antibody binding. Sections were counterstained with haematoxylin, dehydrated in an ascending gradient of ethanol, cleared with xylene, and coverslipped with Consil‐Mount (Thermo Fisher). Representative images containing the area of interest were automatically captured on a slide scanner (Nikon) and exported by using the NanoZoomer 2.0 plug‐in (Hamamatsu) with identical settings for all subjects.

### 2.2 Animals and ethical considerations

Adult male C57BL/6J mice (*n* = 4), Wistar rat dams (*n* = 4, 16 pups of both sex), and *Scgn*‐iCre^BAC^::Ai9 (JAX stock # 007909) reporter mice (*n* = 14) were used (ages: postnatal day [P]1, *n* = 3; P5, *n* = 3; P7, *n* = 3; P11, *n* = 3; P29, *n* = 2). *Scgn*‐iCre^BAC^::Ai9 mice were developed by bacterial artificial chromosome engineering technology^18^, ^19^ Food and water were available ad libitum. Animals were kept under standard housing conditions with a 12 h/12 h light/dark cycle (lights on at 8:00 hours; 55% air humidity). Experiments were approved by the Ethical Review Board of Semmelweis University and the Scientific Ethics Council for Animal Experiments of Hungary (PE/EA/00955‐6/2024), and conformed to the 2010/63/EU European Communities Council Directive. Efforts were made to minimize the numbers of animals and their suffering throughout the experiments.

### 2.3 Single‐cell RNA‐seq

#### 2.3.1 Cell suspension, library preparation, and sequencing

*Scgn*‐iCre::Ai9 mice (*n* = 3, P2) were deeply anesthetized (5% isoflurane in 1 L/min flow‐through air), decapitated, their brains removed, and immersed in ice‐cold pre‐oxygenated (95% O_2_/5% CO_2_) preservation solution containing (in mM) 93 *N*‐methyl‐d‐glucamine‐HCl, 30 NaHCO_3_, 2.5 KCl, 1.2 NaH_2_PO_4_, 20 *N*‐2‐hydroxyethylpiperazine‐*N*‐2‐ethane‐sulfonic acid‐NaOH, 5 Na‐ascorbate, 3 Na‐pyruvate, 0.5 CaCl_2_, 8 MgSO_4_, and 25 glucose (pH 7.4). Cerebral cortices were isolated under microscopy guidance and dissociated in the “papain dissociation system” (Worthington) as per the manufacturer's recommendations with additional mechanical dissociation through Pasteur pipettes with 600, 300, and 150 μm open tips. Cells were resuspended in sterile preservation solution also supplemented with 0.1% bovine serum albumin (BSA), fixed in ice‐cold methanol for 10 min, and stored at −80°C until library preparation. Cells were resuspended in PBS (0.01 M, pH 7.4), followed by their capture, cDNA synthesis, library preparation, and sequencing according to the 10x Genomics Chromium Single Cell Platform. Cells were pooled from all *Scgn*‐iCre::Ai9 mice, with *n* = 9904 cells sequenced and verified by initial quality control.

#### 2.3.2 Reference data and their processing

A molecular atlas of cortical development, for the period of embryonic day 11.5 (E11.5) to P4, served as reference.^20^ Thus, the reference dataset contained *n* = 98 047 cells. These data were subjected to quality control, including the removal of low‐quality cells and doublets. Red blood cells were also omitted. Subsequently, data were normalized, scaled, and reduced in dimensionality using principal component analysis (PCA).^21^ Uniform manifold approximation and projection (UMAP) was trained for the integration and projection of any new data onto the reference embedding, utilizing the first 20 principal components.^22^, ^23^ Dimensional reduction was optimized using the single‐cell dubious embedding detector (scDEED) to determine optimal parameters for a UMAP model with a minimal number of ambiguous cells^24^

#### 2.3.3 Generation of Scgn‐iCre^BAC^:Ai9 dataset

Raw sequencing data were processed with Cell Ranger (v7.0.1). Ambient RNA contamination was mitigated using CellBender (docker://us.gcr.io/broad‐dsde‐methods/cellbender:latest) with a false discovery rate threshold of 0.001.^25^ Cell doublets were identified and removed using Scrublet (v0.2.3).^26^

#### 2.3.4 Quality control

Quality control metrics, including the number of unique molecular identifiers (UMIs), number of genes detected, percentage of mitochondrial and ribosomal gene transcripts, and cell complexity, were assessed. Cells exceeding predefined thresholds for these metrics were excluded, such as total mRNA content (200–10 000 UMIs), gene complexity (0.90), mitochondrial content (<20%), ribosomal content (<2%), and hemoglobin contamination (<0.1%). Pseudogenes, hemoglobin, and ribosomal, sex‐related, and immediate stress response genes were removed when performing feature selection but not from the final matrix. A total of *n* = 7542 cells passed these quality control filters.

#### 2.3.5 Data normalization, dimensionality reduction, and visualization

The filtered dataset was normalized using SCTransform (v0.4.1), regressing out cell complexity and the percentage of mitochondrial and ribosomal gene transcripts. Variable feature selection identified 5000 variable genes, excluding stress response genes, sex‐specific genes, immediate early genes, and housekeeping genes.^27^ Dimensionality reduction was performed using PCA, followed by UMAP (v0.2.2) and *t*‐distributed stochastic neighbor embedding (t‐SNE) (v0.17) and PaCMAP^28^ for visualization. PCA was conducted on variable features, with the first 50 principal components used for downstream optimization using scDEED to determine optimal parameters for UMAP, t‐SNE, and PaCMAP visualizations. Violin plots, dot plots, and heat maps were used to compare gene expression across different cell types and conditions, and visualized in ggplot2 (v3.5.1).

#### 2.3.6 Clustering

Clustering was performed using the Leiden algorithm (v0.4.3.1) across multiple resolutions (0.2–2.0).^29^ Cluster stability was assessed using mrtree (v0.0.0.9000) to identify a robust resolution for the identification of distinct cell populations.^30^ The custom R function was used to guide the selection of resolutions for the final reconciled tree clustering, including a calculation of an adjusted multi‐resolution Rand index, which was then chosen as the maximum value if there was no higher modularity within an additional 0.05 adjusted multi‐resolution Rand index difference.^27^

#### 2.3.7 Cell annotation

Reference‐based annotation was performed by mapping query cells to the reference using 20 dimensions, followed by anchor‐based integration.^31^ Cell‐type labels were transferred from the reference to query cells using the predicted.id scores. The distribution of both *iCre* ^+^ and *tdTomato* ^+^ cells across predicted cell types was analyzed, with particular attention to developmental trajectories. We noted that “deep‐layer corticothalamic (CThPN)” and “subcerebral (SCPN)” but not callosal‐projecting neurons as well as astrocytes to be destinations of *Scgn*‐expressing progenitors and immature neurons coincident with the developmental trajectories of those lineages.^20^ *Hydin* ^+^ and *Dnah12* ^+^ cells (cluster 6) were classified as astrocytes even though they exhibited features more reminiscent of ependymal cells. Cluster 10, classified as “CThPN,” expressed markers such as *Grin3*, *Actn2*, *Tac1*, *Adora2a*, *Drd2*, *Gpr88*, and *Penk*.

#### 2.3.8 Differential gene expression analysis

Differentially expressed genes between clusters were identified using both logistic regression and MAST (v1.30.0) tests.^32^, ^33^ Genes with log_2_ fold change >0.2 (adjusted *p* value <0.001) were only studied further.

#### 2.3.9 Benchmarking and data availability

This study was conducted using the etretiakov/scrna‐seq:jammy‐2024.10.14‐v0.0.3 Docker container, ensuring reproducibility through the workflowr (v1.7.1) framework. All analyses were performed in R (v4.4.1) running on Ubuntu 22.04.5 LTS. Packages utilized include Seurat (v5.1.0.9006), SeuratDisk (v0.0.0.9021), SeuratWrappers (v0.3.5), sctransform (v0.4.1), glmGamPoi (v1.16.0), clustree (v0.5.1), patchwork (v1.3.0.9000), qs (v0.27.2), scCustomize (v2.1.2), mrtree (v0.0.0.9000), scDEED (v0.1.0), tidyverse (v2.0.0.9000), gprofiler2 (v0.2.3), kableExtra (v1.4.0), RColorBrewer (v1.1‐3), viridis (v0.6.5), viridisLite (v0.4.2), skimr (v2.1.5), and foreach (v1.5.2). The complete analysis workflow was made available at https://eugot.github.io/Hanics_2024/01A‐eda.html.

### 2.4 Kainate administration

Kainic acid (Abcam, ab120100) was dissolved in physiological saline at a concentration of 5 mg/mL. Adult male mice (C57BL/6J, *n* = 2/group) were given an intraperitoneal injection of either kainic acid (15 mg/kg) or vehicle (saline), with their behavior regularly monitored for 2 h. All kainate‐injected mice showed epileptic seizures (at least stage 3–4 on a 6‐point Racine‐like scale).^34^, ^35^ Saline‐injected mice behaved normally. Brains were fixed by transcardial perfusion (*see below*) 11 h after the experimental manipulation.

### 2.5 Dark rearing

Wistar rat dams (*n* = 2) were kept in darkness until delivery and throughout the first two postnatal weeks after delivery. Food and water were available ad libitum. Pups of mixed sex were removed at P5, P7, P10, and P14 (*n* = 2/time point), their brains dissected, and immersion fixed in 4% paraformaldehyde (PFA) in 0.1 M PB (pH 7.4). Another dam (*n* = 2) was kept under normal housing conditions, with their offsprings serving as controls (n = 2, mixed for sex, identical developmental time points). On P14, the dams themselves were perfused transcardially and their brains used for immunohistochemistry, too.

### 2.6 Viral circuit tracing

Adult male *Scgn*‐Cre mice (*n* = 2) were mounted in a stereotaxic frame under isoflurane anesthesia (5%, 1 L/min flow rate of tubed air). A Quintessential Stereotaxic Injector (Stoelting) was used to inject pAAV8‐hSyn‐DIO‐mCherry (40 nL/injection; Addgene, #50459) virus particles at a speed of 100 nL/min^36^ in either the dentate gyrus (left hemisphere: anterior–posterior [AP], −1.8 mm; dorsoventral [DV], 2 mm; lateral [L], 1.5 mm from bregma) and/or the barrel cortex (right hemisphere: AP, −1.8 mm; DV, 0.5 mm; L, 3 mm from bregma).^37^ Glass pipettes (Drummond) were withdrawn 5 min after each injection to minimize backflow and off‐target contamination. Mice were transcardially perfused 3 weeks after viral injections, and their brains were processed for immunohistochemistry as below.

### 2.7 Tissue processing for neuroanatomy

Juvenile mice on P1 and P5 were decapitated, their brains rapidly dissected out, and immersion fixed in 4% PFA (wt/vol%) in 0.1 M PB (pH 7.4). Adult mice were deeply anesthetized with a mixture of ketamine (50 mg/kg body weight) and xylazine (4 mg/kg body weight) and perfusion‐fixed transcardially by 4% PFA in 0.1 M PB. Brains were rapidly removed and post‐fixed in the same fixative overnight. Coronal sections (30 μm) were cut on a cryostat after cryoprotecting the brains in 30% sucrose in 0.1 M PB for at least 48 h. A 1‐in‐4 series (inter‐section interval: 120 μm) was used to collect tissues for analysis.

Multiple immunofluorescence histochemistry was performed according to published protocols.^17^, ^38^, ^39^ Free‐floating sections were rinsed in PB (0.1 M, pH 7.4) and pre‐treated with 0.3% Triton X‐100 at 22–24°C for 1 h to enhance the penetration of antibodies. Non‐specific immunoreactivity was suppressed by incubating the specimens in a cocktail of 5% normal donkey serum (NDS; Jackson), 2% BSA (Sigma), and 0.3% Triton X‐100 in PB at 22–24°C for 1 h. Sections were then exposed (at 4°C for 16–72 h) to select combinations of primary antibodies (Table S2) diluted in PB to which 0.1% NDS and 0.3% Triton X‐100 had been added. After extensive rinsing in PB, immunoreactivities were revealed by carbocyanine 2‐, 3‐, or 5‐tagged secondary antibodies raised in donkey (1:200 [Jackson], at 22–24°C for 2 h). Nuclei were counterstained with Hoechst 33342 (1:10 000; Sigma). Sections were dehydrated in an ascending gradient of ethanol, cleared with xylene, and coverslipped with DPX (Sigma). Images were captured on an LSM880 confocal laser‐scanning microscope (Zeiss) with optical zoom ranging from 1 to 3× when using a 40× (Plan‐Apochromat 40×/1.40) objective, with the pinhole set to 0.5–0.7 μm.

### 2.8 In vitro experiments in cell lines and primary cultures

SH‐SY5Y neuroblastoma cells were cultured in Dulbecco's Modified Eagle Medium (DMEM)–GlutaMAX containing 5% foetal bovine serum (vol/vol%), penicillin (100 U/mL), and streptomycin (100 μg/mL; all from Invitrogen) on poly‐d‐lysine (PDL)‐coated six‐well plates at a density of 200 000 cells/well.

Mouse neocortices were isolated on E17, were enzymatically dissociated (trypsin), and plated at a density of 200 000 cells/well on either PDL‐ or laminin‐coated coverslips in 24‐well plates for morphometry. Alternatively, cells at a density of 200 000 cells/well in six‐well plates were used for quantitative polymerase chain reaction (qPCR). Primary cultures were maintained in DMEM/F12 (1:1) containing B27 supplement (2% [vol/vol%]), l‐glutamine (2 mM), penicillin (100 U/mL), and streptomycin (100 μg/mL; all from Invitrogen). Both SH‐SY5Y cells and primary neuron‐enriched cultures were stimulated by 60 mM KCl for 30 min. Cultures were allowed to recover for either 6 h or 24 h, lysed, and collected for qPCR analysis. In another set of experiments, cultures on coverslips were maintained for 72 h, fixed in 4% PFA, and processed for immunocytochemistry as published with select combinations of primary antibodies (Table S2).^40^, ^41^, ^42^

### 2.9 Time‐lapse imaging

*Scgn*‐iCre::Ai9 mouse brains (mixed for sex, *n* = 3, P1) were rapidly dissected on ice, embedded in low‐melt agarose (Sigma), and vibratome‐sectioned (Leica VT1200) coronally at 300‐μm thickness in ice‐cold DMEM containing penicillin (100 U/mL) and streptomycin (100 μg/mL, both from Invitrogen). Acute cortical slices were mounted on Millicell‐CM culture inserts (0.4 μm pore size, 30 mm diameter; Millipore) and equilibrated in Neurobasal‐A medium containing GlutaMAX (2 mM) and 10% foetal bovine serum for 2 h. After 24 h, culture inserts were mounted onto an EVOS FL Auto 2 Microscope (Thermo Fisher) equipped with fluorescence excitation and detection for tdTomato (560 nm), an on‐stage incubator, and tracked by dynamic looped imaging every 60 min.

### 2.10 qPCR

qPCR for *Scgn* was performed on a Bio‐Rad CFX 96 thermal cycler using custom‐designed primers for mouse (*forward*: 5′‐CCC AGA AGT GGA TGG ATT TG‐3′, *reverse*: 5′‐GTT GGG GAT CAG GGG TTT AT‐3′). Protocols, including RNA extraction and cDNA library synthesis, were according to published protocols.^41^ Each sample was run with technical triplicates. Expression levels were normalized to *Gapdh* (*forward*: 5′‐AAC TTT GGC ATT GTG GAA GG‐3′, *reverse*: 5′‐ACA CAT TGG GGG TAG GAA CA‐3′), a housekeeping gene. Melting curves and amplicon sizes were verified.

### 2.11 Statistics for experimental neurobiology

To calculate the ratio of secretagogin^+^ cells in human tissues, immunoreactive versus haematoxylin‐stained somata were counted in the marginal zone (16 000–500 000 μm^2^ frames, *n* = 5/area/section), cortical plate (80 000–1 000 000 μm^2^ frames, *n* = 5/area/section), and subventricular zone (19 000–200 000 μm^2^ frames, *n* = 5/area/section) using the NanoZoomer 2.0 toolbox (Figure S1). Secretagogin^+^ cells in the superior colliculus of mice were counted on five sections/mouse at equal inter‐section intervals to produce equivalent coverage. Fluorescent intensity of calretinin and secretagogin in the dentate gyrus in kainate or vehicle‐treated mice was quantified using the ZEN Software (ZEISS) on an identical number of sections per animal. In in vitro experiments, the length of neurites and secretagogin fluorescence intensity in 50 neurons per condition (KCl vs. vehicle treatment) were measured using the same ZEN Software. A two‐tailed Student's *t*‐test for independent samples was used to evaluate treatment effects. Data were expressed as mean ± SE of the mean. A *p* value of < 0.05 was considered statistically significant.

## 3 RESULTS

### 3.1 Secretagogin expression in the developing human forebrain

**FIGURE 1:**
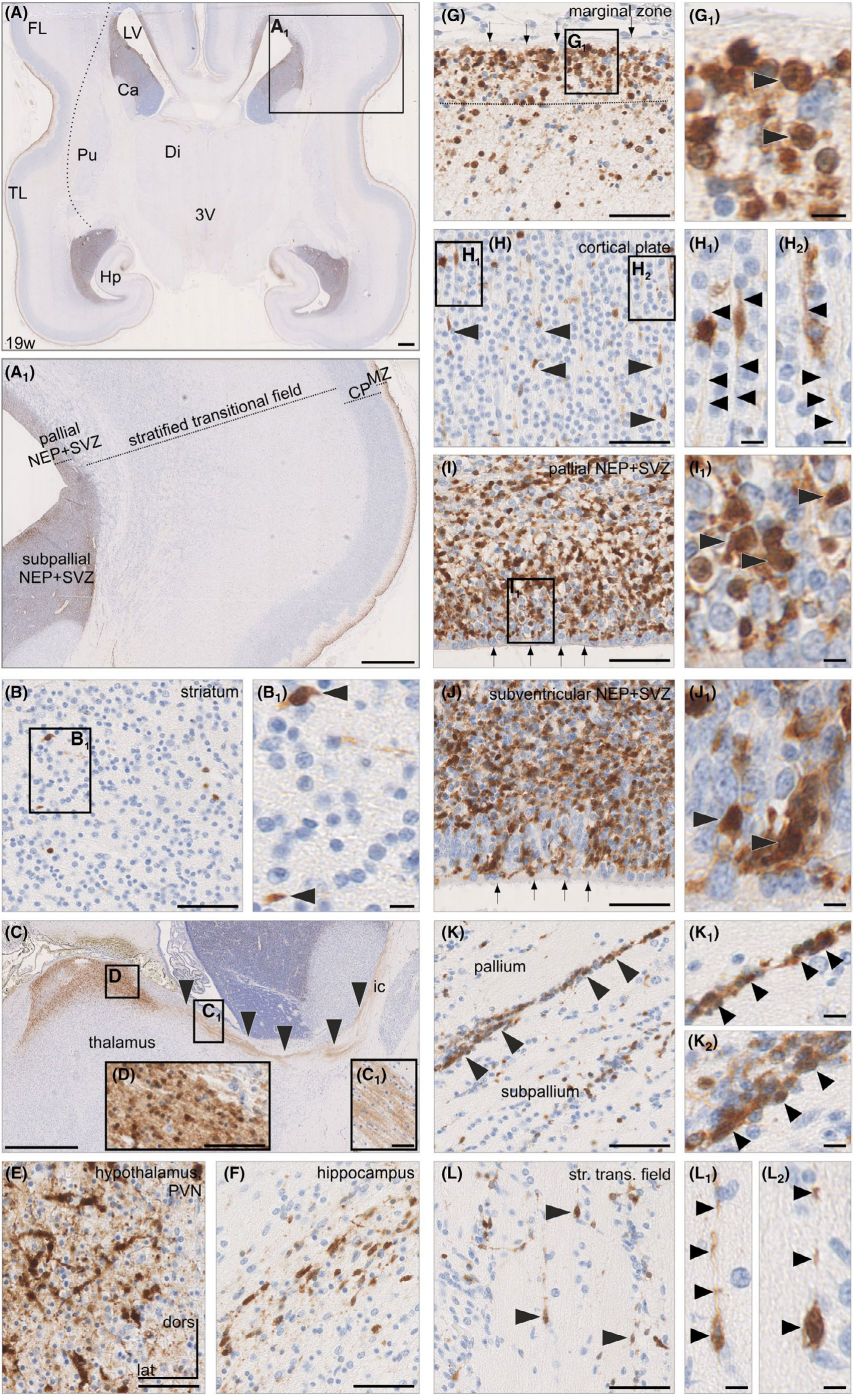
Secretagogin expression in the human foetal telencephalon (GW 19). (A) Overview of a horizontal section. In A_1_, layers of the foetal neocortex are indicated. B,B_1_. Relative paucity of secretagogin^+^ cells in the striatum. C, D. Secretagogin^+^ cells in the dorsal part of the thalamus. E. Secretagogin^+^ cells also populate the paraventricular nucleus of the hypothalamus. F. Bipolar cells in the hippocampus. G,G_1_. Secretagogin^+^ cells (*arrowheads*) in the subpial space (*arrows* point to the pia mater). H‐H_2_. Radially arranged secretagogin^+^ cells (*arrowheads* in H) with bipolar morphology in the cortical plate (*arrowheads* in H_1_ and H_2_ point to processes). I,I_1_. Secretagogin^+^ cells (*arrowheads*) in the neuroepithelium and subventricular zone (*arrows* point to pia mater). J, J_1_. Immunoreactive cells in proliferative zones of the developing caudate nucleus at the ganglionic eminence (*arrows* point to pia mater). K‐K_2_. Tangentially oriented secretagogin^+^ cells at the pallio‐subpallial border. L‐L_2_. Secretagogin^+^ cells in the stratified transitional field of the pallium, the typical area of intense migration. Ca, caudate nucleus; CP, cortical plate; Di, diencephalon; FL, frontal lobe; Hp, hippocampus; ic, internal capsule; LV, lateral ventricle; MZ, marginal zone; NEP, neuroepithelial layer; Pu, putamen; PVN, paraventricular nucleus; SVZ, subventricular zone; TL, temporal lobe. Sections were stained with hematoxylin–eosin. Scale bars = 1 mm (A, A_1_, C), 50 μm (B, G, H, I, J, K, L), 50 μm (C_1_, D_1_, E, F), 10 μm (G_1_, H_1_, I_1_, J_1_, K_1_, L_1_), and 5 μm (B_1_).

Earlier studies used secretagogin to mark thalamocortical afferents^43^ and developing GABA interneurons^14^ in the human foetal brain. Even though these studies were meticulous in their focus on the expression of this Ca^2+^‐sensor protein,^10^, ^17^, ^44^ they have left several cell populations unaccounted for. Particularly, neurons with pyramidal cell‐like arbors in deep cortical layers remain understudied. Therefore, we have performed an unbiased mapping of secretagogin^+^ neurons in the forebrain of human foetuses from early mid‐gestation onward. Secretagogin‐like immunoreactivity was observed in both the neopallium (telencephalon) and archipallium (hippocampus; Figure 1A) during GW 19, particularly in the proliferative zones, as well as the marginal zone of the prospective cerebral cortex. These data do not only corroborate earlier findings^11^, ^14^ but also extend them by identifying secretagogin‐like immunoreactivity in the ganglionic eminences, the striatal proliferative zone, dorsal thalamus, and hypothalamus by mid‐gestation (Figure 1B–E).

Specifically, in the developing neopallium, secretagogin^+^ cells were concentrated in its outermost (Figure 1G,G_1_), intermediate (at the subventricular/subplate boundary; Figure 1H,H_1_), and innermost layers (Figure 1I,I_1_). In the marginal zone (outermost), rows of cells with secretagogin‐like immunoreactivity were seen in the subpial granular layer and in deeper cell rows immediately in the underlying cortical plate. Within the cortical plate, secretagogin^+^ cells had bipolar morphology and were oriented radially (Figure 1H_1_,H_2_). Along the ventricular surface of the telencephalon, both the proliferative (except its innermost stem layer) and the subventricular zones were densely packed with small‐to‐medium diameter cells harboring secretagogin‐like immunoreactivity (Figure 1J,J_1_). Secretagogin^+^ cells also surrounded the pallial–subpallial boundary (Figure 1K–K_2_), as well as the stratified transitional field of the neopallium (Figure 1L).

In the developing archipallium, secretagogin‐like immunoreactivity appeared in the cornu ammonis and decorated granule‐cell‐like cells (Figure 1F) as reported in both primates and rodents.^11^ Cumulatively, these data suggest that neurogenic commitment might coincide with secretagogin expression in the proliferative zones of the human neopallium, with neurons migrating towards the cortical plate and receiving afferentation from, for example, thalamocortical origins, retaining this Ca^2+^‐sensor protein.

### 3.2 Reduced secretagogin expression in Down's syndrome

The distribution of secretagogin‐like immunoreactivity in the human pallium suggests cell fate and/or activity‐dependent protein expression when, for example, cells migrate^41^ or subcortical afferents form^45^ their first synaptic contacts within the subplate and marginal zone.^46^, ^47^ To support the hypothesis that secretagogin could play a role in acquiring architectural features allowing for neuronal activity and/or plasticity,^14^ we have argued that secretagogin expression could be reduced in pathologies associated with a failure of neuronal migration^48^, ^49^ and distorted cortical lamination^50^, ^51^ due to limited intra‐ and subcortical synaptogenesis.^50^, ^51^, ^52^ Down's syndrome is one such inherited debilitating condition.

**FIGURE 2:**
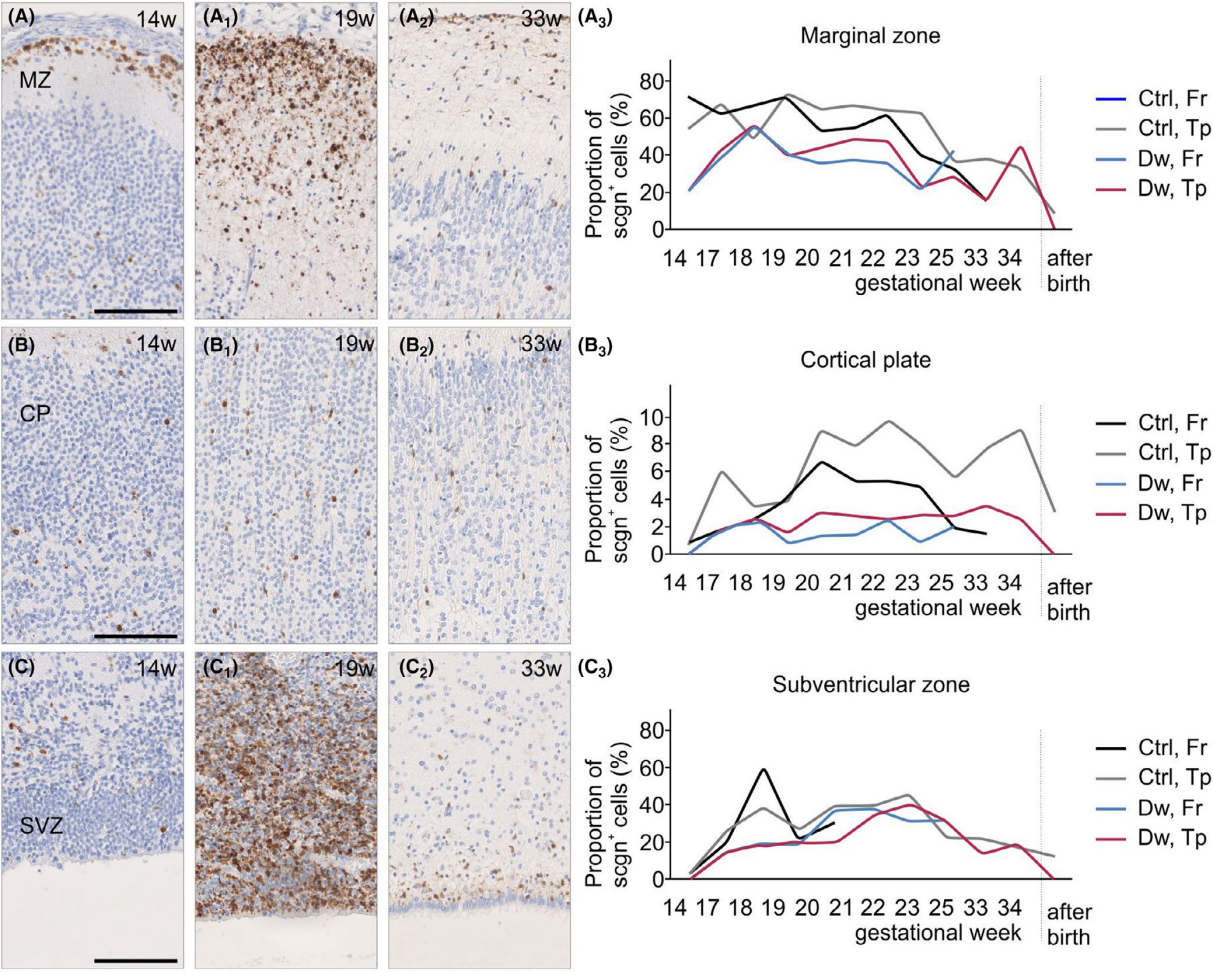
Secretagogin expression in Downs syndrome. Distribution of secretagogin^+^ cells in the marginal zone (A‐A_2_), cortical plate (B‐B_2_), and subventricular zone (C‐C_2_) in control subjects, and their densities compared to those in Down's syndrome subjects (A_3_, B_3_, C_3_) during pregnancy. Secretagogin expression peaked at late mid‐gestation but was reduced in the subventricular zone of the temporal lobe between GWs 18 and 21 and in both the marginal zone and the cortical plate between GWs 20 and 24 in Down's syndrome. Quantitative data show the means of *n* = 2–3 cases/GW to retain visual clarity. CP, cortical plate; Ctrl, control subject; Dw, Down's syndrome subject; Fr, frontal lobe; MZ, marginal zone; SVZ, subventricular zone; Tp, temporal lobe. Scale bar = 50 μm (a–c).

First, we compared the density of perikarya with secretagogin‐like immunoreactivity between cohorts of age‐matched Down's syndrome and control foetuses in the period of GWs 14–34. In the frontal and temporal lobes of control foetuses, secretagogin‐like immunoreactivity peaked in all cortical subfields at late mid‐gestation (i.e., GWs 18–21; Figure 2A–C_2_), with a gradual decline after birth (Figure 2A_3_, B_3_, C_3_). In Down's syndrome, the density of secretagogin‐like immunoreactivity was reduced in the subventricular zone of the temporal lobe between GWs 18–21 (Figure 2C_3_). This was followed by lesser numbers of immunoreactive cells in both the marginal zone and the cortical plate between GWs 20 and 24 (Figure 2A_3_, B_3_). This reduction in the frontal lobe, but not the temporal lobe, was attenuated by GW 24 and persisted until after birth (Figure 2A_3_, B_3_). This chain of events suggests a delay in secretagogin^+^ fate changes and progression along the progenitor, migratory neuroblast, and neuronal levels in Down's syndrome; it also alludes to secretagogin expression undergoing activity‐dependent regulation.

### 3.3 Architectural features of secretagogin^+^ neurons in the postnatal mouse neocortex

**FIGURE 3:**
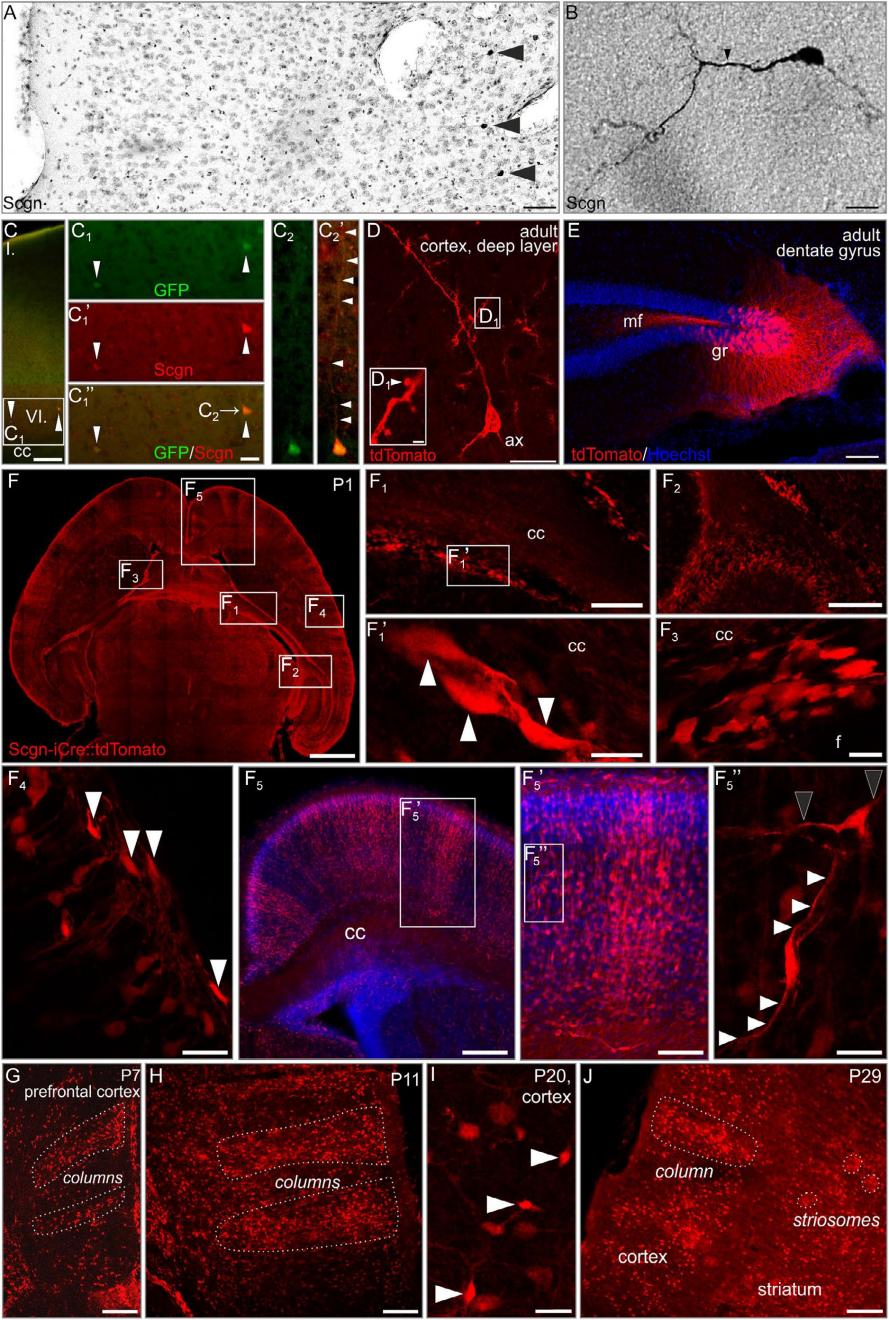
Lifetime tracing of secretagogin expression in the *Scgn*‐iCre::Ai9 mouse brain. A. Overview of the mouse neocortex. Scgn^+^ somata *(arrowheads)* reside in its deep layer, ~150–300 μm apart. B. Scgn^+^ pyramidal neuron in the deep layer of the neocortex. c‐c_2_′. Deep‐layer neocortical neurons show secretagogin immunoreactivity in *Scgn*‐GFP mice. *Arrowheads* in C_1_‐C_1_″ point to GFP^+^/Scgn^+^ somata; arrowheads in C_2_′ indicate the apical dendrite of a deep‐layer pyramidal cell. D. Cortical pyramidal cell labeled in vivo by pAAV8‐hSyn‐DIO‐mCherry virus in *Scgn*‐iCre mice. *Arrowhead* in D_1_ indicates dendritic spines. e. Granule cells of the dentate gyrus and their mossy fibre projection labeled in vivo by pAAV8‐hSyn‐DIO‐mCherry virus in *Scgn*‐iCre::tdTomato mice. F‐F_5_. tdTomato^+^ cells in migratory routes (F_1_, *arrowheads* point to labeled cells), below the pia mater (F_4_, *arrowheads*) and in cortical “columns” (F_5_). Black arrowheads point to horizontally stretching, and *white arrowheads* to vertically stretching processes of labeled cells. G‐J. Columnar organization of the tdTomato^+^ cells is unchanged during the first postnatal month. cc, corpus callosum; gr, granule cell layer; mf, mossy fibres. Scale bars = 1 mm (F), 400 μm (F_5_), 200 μm (F_1_, F_2_), 100 μm (A, C, E, F_5_′, C, G, H, J), 20 μm (D), 10 μm (B, C_1_″, F_1_′, F_3_, F_4_, F_5_″, I), and 2 μm (D_1_).

Recent studies have identified GABA interneurons in the human, primate, and rodent neocortex that express secretagogin.^11^, ^14^ Moreover, early studies in the aged human hippocampus proposed secretagogin expression in both pyramidal^12^, ^53^ and dentate granule cells^11^ (Figure 3A–E), with expression in the latter cell type being experience or activity dependent.^54^ In cerebral organoids, caudal late interneuron progenitor cells also express secretagogin, suggesting interneuron identity.^14^ Nevertheless, non‐interneuron‐like somatodendritic shapes in deep cortical layers (layers 5b and 6a), positioned in a zonal pattern (Figure 3A,C–C_1_″) also exist in rodent cortices. Yet their cellular identity remains unknown.

We have developed *Scgn*‐iCre::Ai9 mice^36^ and studied their cortical tdTomato expression at successive postnatal ages: During the period of P1–P20, tdTomato^+^ cells were organized in single files and associated with the corpus callosum (Figure 3F, F_1_), the fimbrial border of the hippocampal formation (Figure 3F_2_), and contact surfaces between the fornix and corpus callosum (Figure 3F_3_). At the marginal zone, tdTomato^+^ cells had either horizontally flattened somata when apposing the pial surface or round perikarya with a large‐caliber process pointing towards the pial surface (Figure 3F_4_). Within the cortical mantle, tdTomato^+^ cells were observed in column‐like structures with local processes providing connectivity amongst these cells (Figure 3F_5_–F_5_″). Most of these cells had bipolar morphologies, including an ellipsoid body and vertically or tangentially oriented processes. Time‐lapse microscopy confirmed that many of these cells were migratory (Movie S1) and undergoing the expansion of their somata and losing their spindle‐shaped appearance (“flattening”) once becoming stationary (Movie S2). These tdTomato^+^ structures, including cortical ensembles (Figure 3G–I), existed until P29, suggesting the “migrate together wire together” principle as introduced by Morozov and Rakic for cholecystokinin^+^ interneurons.^55^

*Scgn*‐iCre::Ai9 mice allow for the lifetime labeling of the progeny that had undergone recombination during its lifetime. For tdTomato^+^ cells, it is thus not mandatory to continue secretagogin expression postnatally. We have addressed this question by using *Scgn*‐eGFP mice instead, in which the genetic label was co‐stained for secretagogin itself (Figure 3C). While secretagogin expression dissipated in the marginal zone (e.g., in reelin^−^ cells in accordance with^14^), eGFP^+^/secretagogin^+^ deep‐layer neurons with undefined neurotransmitter phenotype(s) remained in the cerebral cortex (Figure 3C–C_2_′). Thus, secretagogin is likely a cell marker that can reveal both permanent and transient expression sites during corticogenesis.

### 3.4 Single‐cell RNA‐seq of tdTomato ^+^ progeny

**FIGURE 4:**
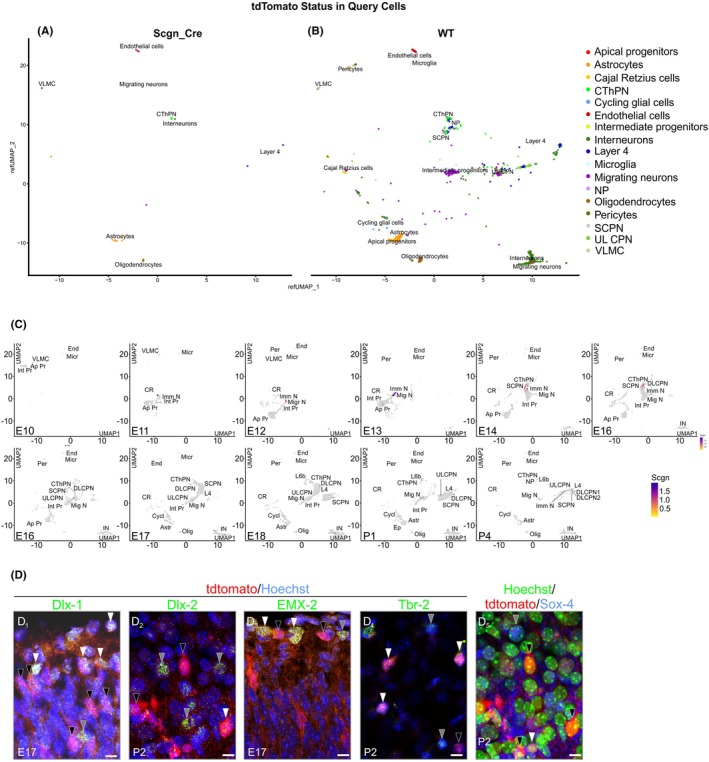
Cellular identity of secretagogin‐expressing cells in the neocortex of infant mice. a,b. Single‐cell RNA‐seq of P2 Scgn‐i*Cre*::Ai9 mouse cortex. UMAP scatter plots with tdTomato^+^/Cre^+^ cells mapped to a reference dataset. *Scgn* was predominantly expressed in migrating and immature neurons. c. UMAP scatter plots with Scgn expression split across developmental stages. Re‐analysis of a reference dataset of cortical development indicated a peak in secretagogin expression between E11 and E15. d. Immunohistochemistry of GABAergic and glutamatergic phenotypes in *Scgn*‐Cre::Ai9 embryonic/early postnatal mouse brains. In all images, *white arrowheads* indicate double‐labeled and *black arrowheads* single‐labeled tdTomato^+^ somata, respectively. *Gray arrowheads* indicate single‐labeled Dlx‐1^+^ (D_1_), Dlx‐2^+^ (D_2_), EMX‐2^+^ (D_3_), Tbr‐2^+^ (D_4_), or Sox‐4^+^ (D_5_) cell bodies. Ap Pr, apical progenitors; CR, Cajal–Retzius cells; CThPN, corticofugal deep‐layer corticothalamic projection neurons; Cycl, cycling glial cells; DL, CPN deep‐layer callosal‐projecting neuron; End, endothelial cells; Ep, ependymal cells; Imm N, immature neurons; IN, interneurons; Int Pr, intermediate progenitors; L4, layer 4 neurons; L6b, layer 6b neurons; Micr, microglia; Migr N, migrating neurons; Olig, oligodendrocytes; SCPN, subcerebral projecting neurons; VLMC, vascular leptomeningeal cells; UL CPN, upper layer callosal‐projecting neuron. Scale bar = 7 μm.

Next, we addressed the identity of cortical progeny that had expressed secretagogin either transiently or permanently. We used *Scgn*‐iCre::Ai9 mice on P2—when maximal expression, including transiently‐labeled cell populations in the marginal zone, interneurons, and deep‐layer neurons are coincidently present—and performed single‐cell RNA‐seq on dissociated cortical cells with or without co‐expression of the bacterial *Ay678269* gene, encoding tdTomato (*n* = 9904). At P2, UMAP scatter plots distinguished 18 major cell clusters, with five clusters containing ~70% of *Ay678269* expression. On the one hand, *Scgn* mRNA was present in progenitors of the astroependymal lineage, also harboring, for example, *Sox4* (49) and *Tbr2* (44). On the other hand, migrating and immature neurons with *Dlx1/2* ^38^, ^39^, ^40^ and *Emx2* (41) were also *Scgn* positive (Figure 4A,B). These data are not entirely unexpected because *Scgn* permanently marks pyramidal cells in human^12^ but not mouse.^56^ However, a unique feature of the rodent brain is that *Scgn* expression vanes in interneurons, while retained in deep‐layer and midline‐associated neurons (for data on *Scgn* in, e.g., indusium griseum, see Refs.^11^, ^54^). Here, we find that among the *Scgn* ^+^ neurons, a significant proportion indeed chose a fate conferring deep‐layer corticofugal projection neuron identity, categorized as “deep‐layer corticothalamic (CThPN) and subcerebral (SCPN) neurons” but not “callosal‐projecting neurons” (Figure 4C) across developmental time‐points. These neurons expressed *Slc17a6* and *Tbr2* mRNA transcripts, confirming their glutamatergic identity and pallial origin. These observations were validated by *Ay678269/*tdTomato^+^ (*Cre* ^+^) expression when overlain on *Scgn* expression in the UMAP of the reference dataset used (Figure 4B). Histochemistry validated the RNA‐seq data by using ortholog markers regionally co‐expressed with *Tbr‐1*, *Dlx1*, and *Sox11* ^57^, ^58^, ^59^ revealing SCGN/Sox4, SCGN/Dlx1/2, SCGN/Tbr‐2, and SCGN/Emx2 co‐existence (Figure 4D). Lastly, we injected pAAV8‐hSyn‐DIO‐mCherry particles in the neocortex of *Scgn*‐iCre mice,^18^ which revealed pyramidal‐shaped neurons with axons leaving the neocortex (Figure 3D) in the corpus callosum, providing anatomical verification for principal cell identity. Overall, these findings do not only corroborate our neuroanatomical mapping in human and mouse but define a novel neuronal subtype destined to deep cortical layers. The presence of *Scgn* in cycling progenitors and distinct lineages of neurons—transiently or permanently—suggest that *Scgn* could be a “use‐dependent” or “cell state‐dependent” molecular feature and as such sensitive to environmental manipulations during neuronal morphogenesis.

### 3.5 Excitation increases secretagogin expression

**FIGURE 5:**
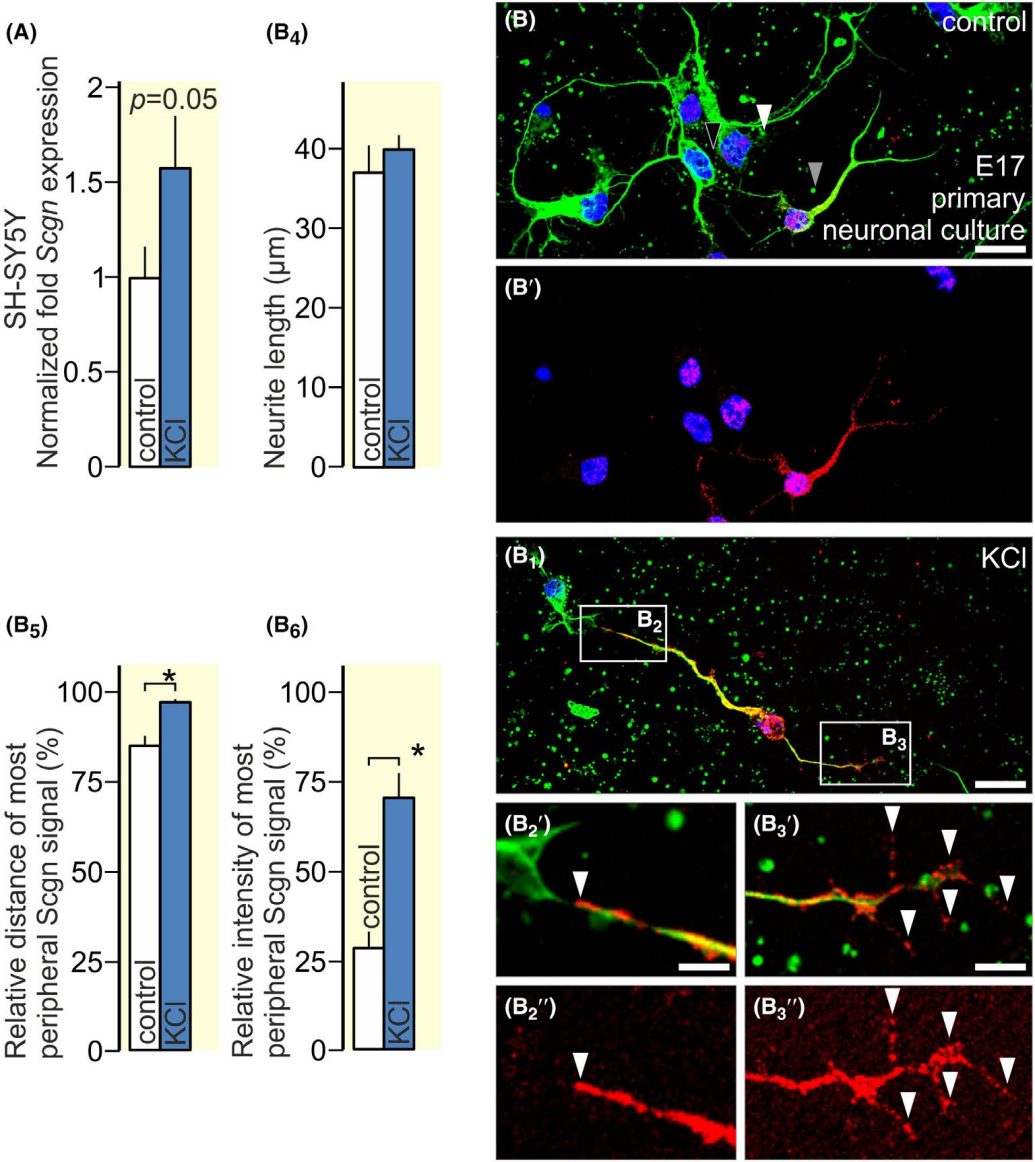
Secretagogin expression modulated by KCl in vitro. A. KCl (60 mM) increased secretagogin transcription in SH‐SY5Y neuroblastoma cells. B‐B_3_. KCl (60 mM) increased secretagogin expression, particularly in distal neurites and putative growth cones (*arrowhead* in B_2_, B_2_′) and in filopodia (*arrowheads* in B_3_, B_3_′) in primary neuronal cultures. B_4_–B_6_ KCl increased the length of secretagogin immunoreactivity in distal neurites including growth cones. Scale bars = 10 μm (B, B_1_,) and 3 μm (B_2_′, B_3_′). *(*p* < 0.05).

Firstly, SH‐SY5Y neuroblastoma cells were exposed to KCl (60 mM) for 30 min to model pharmacologically induced activity. KCl increased *Scgn* mRNA transcript levels (*p* < 0.05; Figure 5A). Likewise, KCl stimulation led to the translocation of secretagogin to neuronal processes in primary cultures, with a remarkable increase in the protein in distal‐most neurites, including growth cone‐like structures (Figure 5B–B_3_″). Quantitative analysis substantiated increased secretagogin immunoreactivity in both the length and density of immune‐positive neurite segments (Figure 5B_4_–B_6_).

**FIGURE 6:**
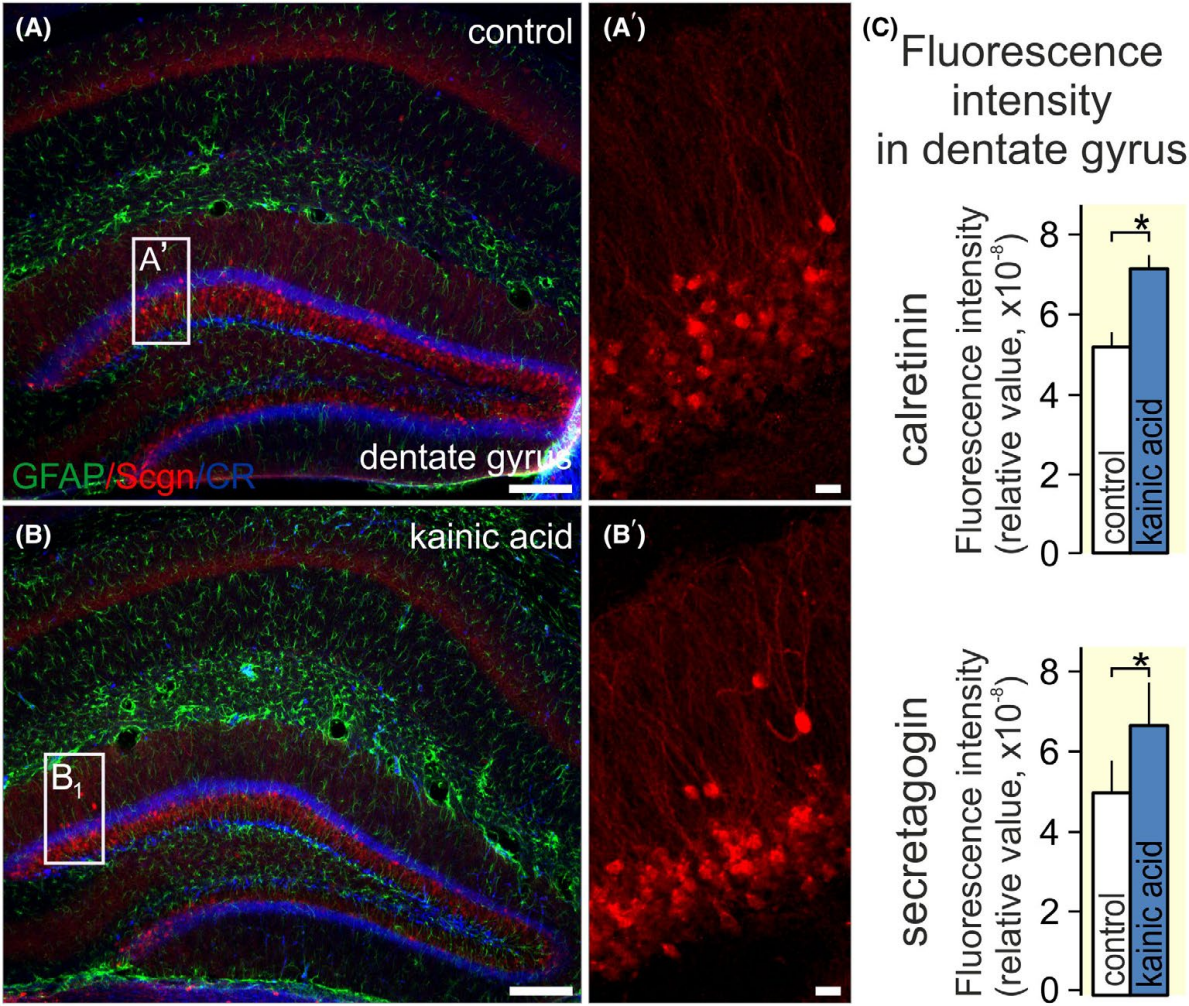
Secretagogin immunoreactivity in the dentate gyrus upon systemic kaniate exposure. A–C. Intraperitoneal kainate administration coincidently increased calretinin and secretagogin immunoreactivity in mouse dentate gyrus. Secretagogin was localized to granule cells. Scale bars = 100 μm (A, B) and 10 μm (A′, B′). *(*p* < 0.05).

Secondly, we hypothesized that, analogous to KCl, systemic kainate treatment known to induce epileptiform activity in the hippocampus^34^ could affect secretagogin expression. Eleven hours after intraperitoneal kainate application (15 mg/kg), both calretinin (7.12 ± 0.35 [kainic acid] vs. 5.21 ± 0.32 [no treatment], arbitrary units [a.u.]; *p* < 0.05) and glial fibrillary acidic protein (GFAP) immunoreactivities increased in the dentate gyrus,^39^, ^60^ validating the drug effects (*n* = 2/group; Figure 6A–C). Simultaneously, kainate increased secretagogin immunoreactivity in dentate granule cells (Figure 6A–C, 6.77 ± 1.135 a.u. [kainic acid] vs. 5.13 ± 0.78 a.u. [no treatment]; *p* < 0.05). These data suggest that secretagogin expression and subcellular distribution are sensitive to stimuli that increase neuronal excitability.

### 3.6 Dark rearing reduces secretagogin expression in the rat visual system

**FIGURE 7:**
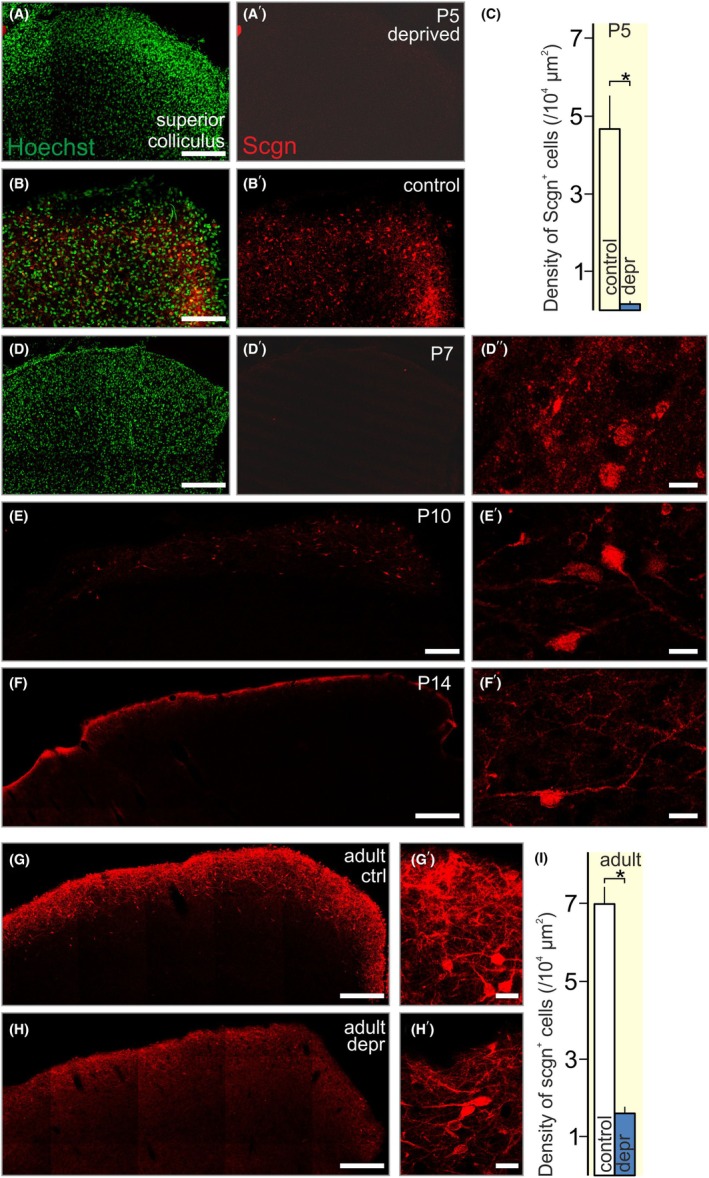
Loss of secretagogin expression in the colliculus superior upon light deprivation. A–C. Secretagogin was not detected in the colliculus superior pf dark‐reared rat pups on P5 versus controls kept under a normal light cycle. D–F. In dark‐reared pups, secretagogin expression remained retarded at P7 and only detectable to P10 with a further decline at P14. G–I. In the dams' brains, the number of secretagogin^+^ neurons in the superior colliculus also decreased if the rats were kept in darkness for 14 days. ctrl, control; depr., light‐deprived. Scale bars = 200 μm (A, B), 100 μm (D–H), and 10 μm (D″, E′, F′, G′, H′). *(*p* < 0.05).

The visual system relies on sensory input (i.e., light) for its wiring and experience‐dependent maturation, with the most critical period for neuronal specification from before eye opening to over the first postnatal month in rodents.^61^, ^62^, ^63^ The core circuit layout includes retinal ganglion cell axons to innervate retinorecipient nuclei, such as the superior colliculus and lateral geniculate nucleus, which then send axons to the visual cortex, as well as feed chronospecific circuits of the hypothalamus.^64^ Notably, each module of the visual system contains secretagogin‐expressing neurons in vertebrates.^11^, ^65^, ^66^, ^67^ Particularly pronounced secretagogin immunoreactivity characterizes neurons of the superior colliculus, the major relay of the tectofugal visual pathway.^11^, ^66^ Dark rearing, when rodent offspring are deprived of light during the critical period of visual system plasticity, is a common approach to disrupt the activity‐dependent development of the visual system.^68^, ^69^ Here, rat dams delivered and reared their offspring in complete darkness. Subsequently, their brains were dissected and analyzed for secretagogin expression. Dark rearing induced the delay of secretagogin expression in pups as early as P5 (*p* < 0.05; Figure 7A–C). Secretagogin immunoreactivity remained undetectable or minimal in light‐deprived offspring until P14 (Figure 7D–F). Simultaneously, the dams' colliculi underwent reorganization and lost secretagogin expression in adulthood (Figure 7G–I). These data support that secretagogin is sensitive to both gain‐ and loss‐of‐function manipulations, reinforcing that secretagogin expression is regulated activity dependently during postnatal development.

## 4 DISCUSSION

In this study, we introduce secretagogin as an activity‐dependent and transiently expressed EF‐hand Ca^2+^‐binding protein in the developing forebrain. We base this conclusion on (i) the systematic neuroanatomical analysis of the human foetal brain, (ii) neuroanatomical and single‐cell profiling of secretagogin^+^ neurons in transgenic mice, and (iii) in vitro and in vivo experiments, which support that intrinsic neuronal activity promotes the expression of secretagogin.

Previous advances using integrative gene co‐expression analysis identified secretagogin only in GABA interneurons, with their origin in the caudal and lateral ganglionic eminences.^14^ Later, secretagogin was shown in four major sources of GABAergic neurons, including the caudal and lateral ganglionic eminences, the subpallial septum, and the preoptic area.^70^ In contrast to these findings, which described secretagogin expression only in interneurons in early foetuses and at mid‐gestation,^14^, ^70^ sporadic data suggest that secretagogin can also be expressed in glutamatergic neurons, too.^12^ Changes in secretagogin expression in Alzheimer's disease^12^ lend support to the possibility of activity‐dependent regulation for this protein.

To interrogate secretagogin expression sites, we performed a systematic neuroanatomical study in the developing human foetal brains, from GW 14 until birth. Secretagogin expression was found to peak at mid‐gestation, persist during the third trimester with a decline just before birth.^70^ Notably, secretagogin expression was found to be transient in superficial layers of the developing cerebrum and even in subcortical regions (e.g., striosomes, *data not shown*). It is therefore tempting to speculate that secretagogin participates in shaping Ca^2+^‐dependent intracellular mechanisms (e.g., growth cone navigation, lead process expansion) particularly relevant to motile cell contingents in the marginal zone of the cerebral cortex. Interestingly, secretagogin^+^ neurons in the marginal zone lacked *Reln*, encoding reelin, but contained *Dly1/2* mRNA in mice, thus alluding to cellular heterogeneity beyond classical Cajal–Retzius cell identity focally. Thus, prospective interneurons could transiently express secretagogin in superficial layers of the human neocortex.^71^

The temporal dynamics of secretagogin expression in human foetuses allude, even if indirectly, to cell state‐specific regulatory mechanisms. To address this hypothesis, we have developed *Scgn*‐iCre::Ai9 mice, an experimental life‐long cell‐tagging genetic approach, because tdTomato is expressed in adult offspring regardless of when the *Cre*‐mediated excision of stop codons occurred to allow for tdTomato expression. Thus, *Scgn*‐iCre::Ai9 mice might faithfully reveal the sum of transient and permanent secretagogin expression sites. Here, the accumulation of tdTomato signal in interconnected cortical neurons arranged vertically in “column‐like structures” alluded to expressional up‐regulation upon the activity‐dependent intracortical wiring. Besides, dual‐label histochemistry for tdTomato/secretagogin identified bona fide secretagogin^+^ neurons in the adult mouse forebrain. This approach contrasted data from *Scgn*‐GFP mice in which GFP expression was directly dependent on the acute activity of the *Scgn* minimal promoter sequence, assuming sufficiently tight genetic control. Indeed, GFP expression coincided with the histochemically verified presence of secretagogin. The subsequent combination of single‐cell RNA sequencing, *post‐hoc* immunohistochemistry, and viral tracing in *Scgn*‐Cre mice demonstrated that deep‐layer cortical neurons survive and retain secretagogin expression postnatally and establish long‐range efferents toward subcortical targets. These data cumulatively identify transient and permanent secretagogin expression patterns in the nervous system.

Next, we aimed to substantiate if secretagogin expression could be activity dependent, alike in the pancreas. First, we performed the comparative analysis of Down's syndrome cases during foetal development and at birth (vs. age‐matched controls). We were motivated by the fact that Down's syndrome is characterized by multiple developmental deficits of the telencephalon, including errant cell migration^48^, ^49^ and impaired cellular morphogenesis, prominently delayed synaptogenesis.^50^, ^51^, ^52^ More specifically, the proportion of calretinin over calbindin in GABA neurons is reduced in Down's syndrome.^48^ Lamination defects are also reflected by the altered distribution of subsets of interneurons harboring Ca^2+^‐binding proteins.^72^ However, parvalbumin, calbindin‐D28k, and calretinin differ from members of the S100 protein family because they are cytosolic proteins not released in mammals (unlike in snakes). This difference explains why S100 proteins, many expressed by astrocytes, could promote cell survival during development or injury^73^ or shape cytoskeletal growth, which impacts neural differentiation.^74^ Moreover, secretagogin is implicated in modulating neuronal migration^41^ and neuronal‐^14^ and synaptic^45^ morphogenesis. Thus, reduced secretagogin levels in Down's syndrome cases, relative to age‐matched controls, could suggest the slowed maturation of neuronal circuits in Down's syndrome, an observation compatible with the hypothesis of activity‐dependent gene regulation.

Thereafter, we used in vitro and in vivo experiments to stimulate or dampen cellular plasticity. A consensus outcome of these experiments is that (i) excess excitation increases secretagogin levels in neurons; (ii) deprivation of circuit activity occludes secretagogin expression, which otherwise parallels the physiological assembly of neuronal networks during brain development; and (iii) secretagogin expression is diminished in adult neuronal circuits deprived of afferent (visual) stimuli. This evidence underpins the hypothesis that transient secretagogin expression in neurons could be dependent on neuronal activity. Cumulatively, our data suggest that secretagogin has use‐dependent and thus, labile, expression in the central nervous system and is associated with cellular states (nucleokinesis, morphogenesis, sensory modulation, and neurotransmission^45^) of high energy demand and/or profound Ca^2+^ signaling and dependence. Given that the secretagogin interactome includes many ligand‐gated ion channels, molecular components of the axonal transport, and SNAP Receptors machineries,^18^, ^36^ we suggest that transient waves of secretagogin expression could allow this protein to function as a Ca^2+^‐dependent coincidence detector and/or actuator in physiological processes.

## AUTHOR CONTRIBUTIONS

**János Hanics:** Investigation. **Evgenii O. Tretiakov:** Investigation. **Roman A. Romanov:** Investigation. **Anna Gáspárdy:** Investigation. **Zsófia Hevesi:** Investigation. **Robert Schnell:** Investigation. **Tibor Harkany:** Conceptualization; funding acquisition; writing – original draft; writing – review and editing; supervision; resources. **Alán Alpár:** Conceptualization; funding acquisition; investigation; writing – original draft; writing – review and editing; supervision.

## CONFLICT OF INTEREST STATEMENT

The authors declare that they have no conflicts of interest.

## Supporting information


Data S1.

Movie S1.

Movie S2.

## Data Availability

All data pertinent to anatomical findings in this study were presented in either the core figures or SI. Data on single‐cell transcriptomics were made available on GitHub (https://eugot.github.io/Hanics_2024/01A‐eda.html).
