# Supplementary material for: Neuronal activity modulates the expression of secretagogin, a Ca2+ sensor protein, during mammalian forebrain development
Source: Acta Physiol (Oxf). 2025 Mar 31;241(5):e70031. doi: 10.1111/apha.70031 (PMC11959173; doi:10.1111/apha.70031)
Supplement: Supplementary file 1 — Data S1. [file APHA-241-e70031-s002.docx]

**Supplementary information to:**

**Neuronal activity modulates the expression of secretagogin, a Ca^2+^ sensor protein, during mammalian forebrain development**

**János Hanics^1,2^, Evgenii O. Tretiakov^3^, Roman A. Romanov^3^, Anna Gáspárdy^1^, Zsófia Hevesi^3^, Robert Schnell^3,4^, Tibor Harkany^3,4,^* and Alán Alpár^1,2,^***

*^1^Department of Anatomy, Semmelweis University, Budapest, Hungary;*

*^2^SE NAP Research Group of Experimental Neuroanatomy and Developmental Biology, Semmelweis University, Budapest, Hungary;*

*^3^Department of Molecular Neurosciences, Center for Brain Research, Medical University of Vienna, Vienna, Austria,*

*^4^Department of Neuroscience, Biomedicum 7D, Karolinska Institutet, Solna, Sweden.*

**^*^Corresponding author:** Dr. **Alán Alpár***,* H-1085 Budapest, Tűzoltó utca 58., Budapest, Hungary

*Tel*: +36 1 2156 920 / 53609, *Fax*: +36 1 2155 158

*e-mail*: [Alpar.Alan@semmelweis.hu](mailto:Alpar.Alan@semmelweis.hu)

**Dr. Tibor Harkany**, A-1090 Vienna, Spitalgasse 4, Vienna, Austria

Tel: +43 1 4016034050 / 34051

e-mail: [Tibor.Harkany@meduniwien.ac.at](mailto:Tibor.Harkany@meduniwien.ac.at) or [Tibor.Harkany@ki.se](mailto:Tibor.Harkany@ki.se)


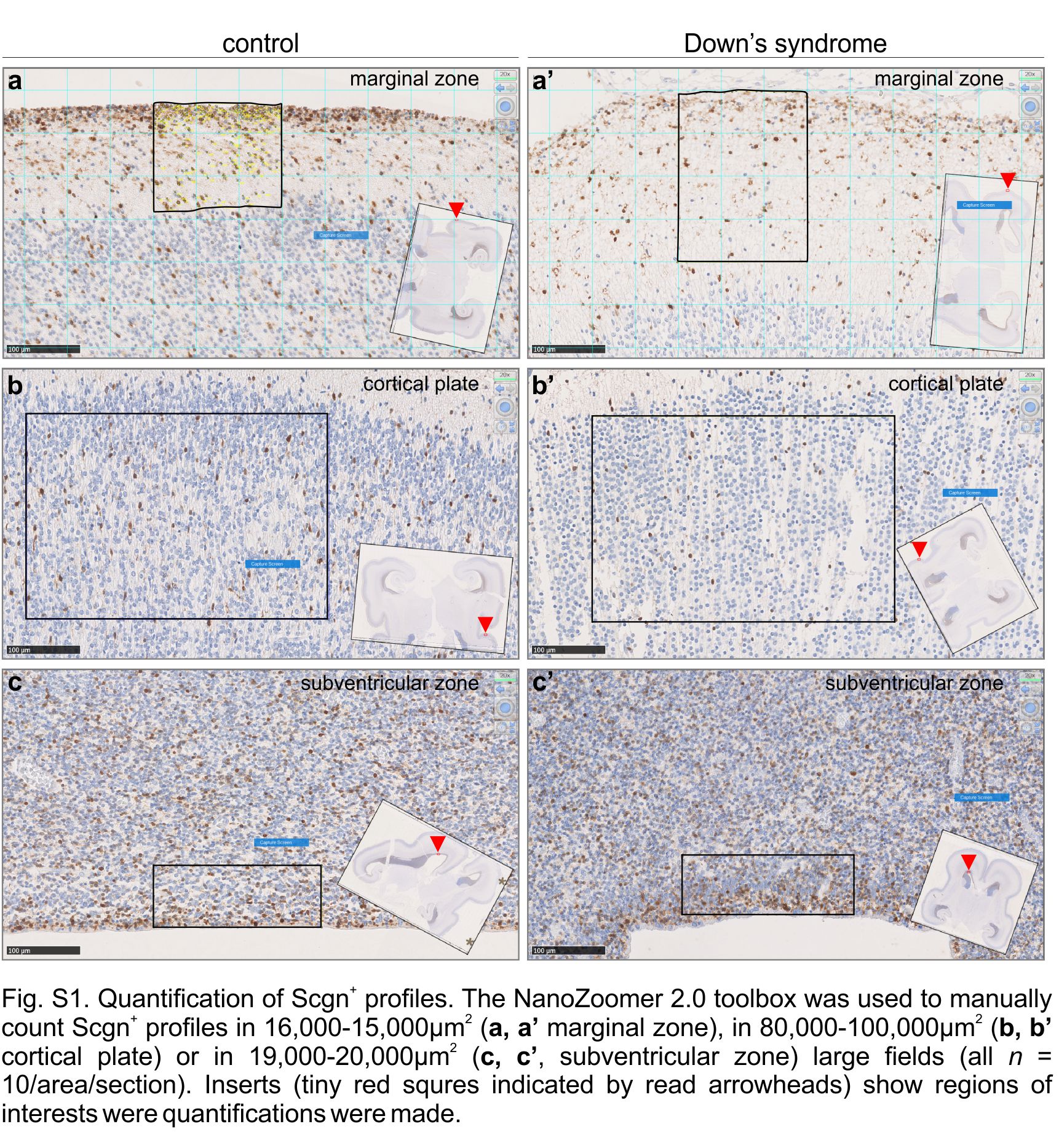


**Fig. S1** – Quantification of secretagogin^+^ profiles.

The NanoZoomer 2.0 toolbox was used to manually count secretagogin^+^ profiles in 16,000 by 15,000 μm^2^ (**a,a’** marginal zone), in 80,000 by 100.000 μm^2^ (**b,b’** cortical plate) or in 19,000 by 20,000 μm^2^ (**c,c’** subventricular zone) sized fields (*n* = 10/area/section). Inserts show regions of interest with red arrows pinpointing the location of the high-power images in which quantification was performed.

**Table S1** – Human fetal subjects used in this study.

| **ID** | **Age in weeks includes gestational period** | **Age in days includes gestational period** | **Sex** | **Type** |
| --- | --- | --- | --- | --- |
| 240-11  232-09  056-11  033-11  178-10  195-11  104-11  131-11  029-12  074-11  151-11  039-11  184-10  192-11  149-10  236-11  127-11  216-09  216-11  128-11  013-11  199-11  207-10  086-10  072-09  054-10  040-11  184-08  06810  169-09  067-09  194-09  050-05  061-12  066-09  004-09  090-08  147-05  095-10  118-07  041-11  224-11  036-11  119-04  060-05  141-09  091-06  047-02  239-08  053-01  229-08  228-11 | 14  14  15  15  15  16  17  17  18  19  19  19  19  20  21  21  22  22  22  22  23  23  26  27  28  33  34  42  49  14  16  16  17  18  19  19  19  20  20  21  22  22  22  22  23  23  23  25  33  34  34  53 | 101  102  105  106  108  118  119  125  131  133  136  137  137  146  148  149  154  158  158  159  161  163  182  190  197  235  242  299  349  099  111  112  116  126  130  131  135  138  140  145  151  154  156  157  158  161  162  173  231  235  236  369 | m  nn  f  f  nn  nn  nn  f  m  nn  m  f  m  m  f  m  m  m  m  m  f  nn  m  m  f  f  f  m  f  nn  f  nn  f  m  f  m  f  m  m  m  m  f  m  m  m  f  m  f  m  m  m  m | control  control  control  control  control  control  control  control  control  control  control  control  control  control  control  control  control  control  control  control  control  control  control  control  control  control  control  control  control  Down’s syndrome  Down’s syndrome  Down’s syndrome  Down’s syndrome  Down’s syndrome  Down’s syndrome  Down’s syndrome  Down’s syndrome  Down’s syndrome  Down’s syndrome  Down’s syndrome  Down’s syndrome  Down’s syndrome  Down’s syndrome  Down’s syndrome  Down’s syndrome  Down’s syndrome  Down’s syndrome  Down’s syndrome  Down’s syndrome  Down’s syndrome  Down’s syndrome  Down’s syndrome |

*Abbreviations*: f, female; m, male; nn, not known.

**Table S2** – List of markers used for immunolabelling

| **Marker** | **Source** | **Host** | **IH dilution** | **Reference** |
| --- | --- | --- | --- | --- |
| β-III-Tubulin (TUJ1) | Sigma | mouse, mc^1^ | 1:2,000 | Hanics et al., 2017 |
| Calretinin | Swant | mouse, mc^1^ | 1:1,000 | Alpár et al. 2010 |
| Dlx-1 | Atlas Antibodies # HPA 045884 | rabbit, pc^2^ | 1:200 | this study |
| Dlx-2 | Atlas Antibodies # HPA 056965 | rabbit, pc^2^ | 1:200 | this study |
| EMX-2 | Atlas Antibodies # HPA 065294 | rabbit, pc^2^ | 1:50 | this study |
| GFAP | Synaptic Systems | guinea pig, pc^2^ | 1:1,000 | Antonucci et al. 2012. |
| SOX-4 | Atlas Antibodies # AMAb 91378 | mouse, mc^1^ | 1:1,000 | this study |
| Tbr-2 | Atlas Antibodies # HPA 028896 | rabbit, pc^2^ | 1:200 | this study |
|  |  |  |  |  |
| Secretagogin | kind gift from L. Wagner | rabbit, pc^2^ | 1:5,000 | Wagner *et al.*, 2000 |

^1^monoclonal antibody, ^2^polyclonal antibody
